# Error in Author Name

**DOI:** 10.1001/jamanetworkopen.2026.36706

**Published:** 2026-09-03

In the Original Investigation titled “Global and Domain-Specific Cognitive Outcomes After Catheter Ablation for Atrial Fibrillation,”^1^ published July 31, 2026, Drs Triantafyllou's and Bakogiannis’s first names were misspelled in the byline. The article has been corrected.^1^
